# Address before the International Hahnemannian Association

**Published:** 1888-08

**Authors:** Wm. P. Wesselhœft

**Affiliations:** Boston, President


### HOMŒOPATHIC PHYSICIAN,

A MONTHLY JOURNAL OF

HOMŒOPATHIC MATERIA MEDICA AND CLINICAL MEDICINE.

"If our school ever gives up the strict inductive method of Hahnemann, we are lost, and deserve only to be mentioned as a caricature in the history of medicine."—CONSTANTINE HERING.

Vol. VIII. AUGUST, 1888. No. 8.

### ADDRESS BEFORE THE INTERNATIONAL HAHNEMANNIAN ASSOCIATION AT ITS NINTH ANNUAL MEETING.

DR. WM. P. WESSELHŒFT, BOSTON, PRESIDENT.

*Members of the International Hahnemannian Association:*

On this occasion, on the ninth meeting of this Association, our thoughts naturally revert to the events which have occurred since our last gathering and which most deeply interest and affect our organization. We have met with a deep bereavement in the death of two of our most valued and zealous members. The one was taken from his field of labor after having passed the allotted time of life; the other was stricken suddenly in the full vigor of ripe manhood.

Both men were as true as steel to their convictions, and of both we may say: "Well done, good and faithful servant."

In the death of Dr. Lippe not only this Association but the entire profession have lost a leader, a master, and an uncompromising worker. The city of Philadelphia, in which the greater part of his professional life was spent, has lost one of the greatest healers since the light of Hahnemann came upon earth. Hundreds could surpass him in physical diagnosis and pathology, and demonstrate their sagacity at the autopsy, but none could approach him in the diagnosis of the indicated remedy, and for this reason autopsies were fewer in his practice!

He was ever watchful of the retrograde tendencies in the practice of the so-called homœopathists, and never lost an occasion to express his convictions and defend the strictest methods in the treatment of the sick. With what admirable courage, power, and effective argument he met his adversaries all of us are familiar and thankful. His therapeutical success made him bold and confident, and at the same time most intolerant of practices which could only lead to failures. The cures he published were always instructive, never failing to give good reasons for the selection of the drug, and teaching us the necessity of waiting for its full action, and not carelessly to repeat a well-selected remedy. The most important book he has left us is, in my judgment, *The Key to the Materia Medica*, published in 1854, and which contained a study of a dozen polychrests, with concordant symptoms of over three hundred other drugs.

This little work of one hundred and forty-four pages contains a mine of wealth, and as a guide to the materia medica is unsurpassed by any other repertory, although arranged on an entirely different plan from those mostly in use. Its scope is so large that it should have a place on the desk of every Hahnemannian, and as it is out of print, our Association ought to see to it that every member has a copy. It vies in usefulness with Bœnninghausen's *Therapeutic Pocket Book*, and will be found of priceless value to him who prefers to prescribe for the complex of symptoms rather than for a pathological lesion alone.

Dr. Lippe's strict integrity in his practice challenged the admiration of that small portion of the younger men of our school who were striving to learn something better than the worse therapy taught in most of the colleges, and to such he was an ever ready teacher and adviser at all times, willing to show them that Hahnemann's statement was correct when he said: "Besides pure Homœopathy, another true and more perfect way of healing dynamic (*i. e.*, non-surgical) diseases cannot exist, as it is impossible to draw more than one straight line between two given points." How little must he who imagines there are still other kinds of diseases to be cured besides those amenable to Homœopathy, have penetrated into its depth, or how insufficiently must he have practiced it; how few correctly planned homœopathic cures must he have seen or read of, and, on the other hand, how imperfectly must he have weighed in his mind the absence of any foundation in every allopathic mode of procedure or have acquired information concerning their poor, nay, horrible results; who, with shallow indifference, considers the merits of the only true healing art as equal to those pernicious modes of treatment, or who even pretends that they are the sisters of Homœopathy, and indispensable to it. My true and conscientious followers may refute such notions by their almost unerring fortunate cures.

Dr. Lippe did refute such notions, and by ocular demonstration guided his students into safer fields of labor, in which their enthusiasm for law and method will grow from year to year, as his own did.

Unlike those among us who are ready and eager to adopt any and every means for "relieving pains" or suppressing symptoms, and who throw overboard four-fifths of the materia medica, and, consequently, use in their practice one-fifth of it, he taught and made it evident that the whole materia medica can be used to advantage if put into proper requisition, and that the greater the range of medicines used (which means only closer individualizing) the greater the success.

We now come to the consideration of other matters affecting our small and protesting organization. We are met by attacks and criticisms from the homœopathic world at large, which bear the closest resemblance to the assaults our fathers had to meet from the allopathic world more than fifty years ago. We read in the journals of "the little exclusive coterie," "the bottle washers," "the idolators of the master," and what not more. Some even go so far as to accuse us of wrong doing in ceasing to act like a wholesome leaven to the ponderous American Institute of Homœopathy.

Let us look at the facts: Nine years ago a dozen men convened in this same place to form an association which should represent Homœopathy and all its great truths. This step was not taken hastily or rashly, but with very good and sufficient reason. The American Institute of Homœopathy had stricken from the requirements of membership the word "Homœopathy," and thereby opening its doors to any man or woman who possessed a diploma, not even requiring a *quasi* adherence to, much less a knowledge of, the new healing art. Graduates of any medical college were admitted to full membership, and permitted to participate in its deliberations. The flood-gates were opened, and all comers with diplomas were sucked into the wide sluiceway. It was proclaimed that this Institute recognized no distinction among physicians; anybody with a diploma was eligible. This great and liberal measure was enacted in 1874, mainly through the instrumentality of a few men who had placed themselves on record a year or two previous as champions of the single remedy in its high attenuation.

The wretched result of this action has become more and more evident from year to year, the papers, discussions, and actions taken by this so-called Homœopathic Institute clearly showing a steady departure from any kind of method and law until the little leaven remaining became incapable of raising such dough. The yeast of the American Institute of Homœopathy became sour after six years of effort, and very naturally felt a desire to regain its "raising" qualities by starting a new bake shop in which only honest materials were to be used, and which would act readily to the best advantage of the loaf. When the advice is given us by our kind *medical counselors* not to "flock by ourselves," but to remember the biblical assertion that "a little leaven leaveneth the whole lump," they forgot the ingredients and component parts of the lump. When you take dough, constituted of meal and water, and add a little leaven it will rise, but when the dough is composed of a conglomerate mass which can never be converted into chyme by the most approved stomach ever constructed, we have a right to suspect the composition of the lump to be at fault and not the leaven. Let us follow this *simile*, which has been forced upon us and is not of our own making, a little further: Suppose a baker who engages to deliver good bread, and into the composition of which only pure and honest material is to be put, allows his associates to throw handfuls of extraneous substances into the dough in order to increase its weight and bulk, the result will be that his customers will soon recognize the fraud, and the few honest journeymen among his employees will seek work in another shop. Can an association which meets annually and has a session of four days undertake to teach *an art* to men and women who have never found it convenient to acquaint themselves with its possibilities by study and experience? The art represented by the American Institute of Homœopathy may be able to do it, but the work of Hahnemann requires a worthier candidature.

The Institute may take it for granted that no one will apply for membership who is not in sympathy with the law of similars in the treatment of the sick. This may be true or not; the fact remains that anybody in possession of a diploma, regardless of his views, experiences, or convictions, will be welcomed with open arms.

And for what purpose do they come? This question the circular answers most alluringly, "that each may labor for his or her own favorite branch of study." Now, as Homœopathy is a system of therapeutics only, based *not only* upon the similar remedy, but quite as much on the single remedy and the dynamized dose, we have a right to expect that the therapeutics of Homœopathy be not only discussed, but evidence of their application given in the various departments of an ostensibly homœopathic society. Please take down the last volume of the *Transactions of the A. I. H.* and look at the work of the different bureaus, especially the gynæcological report and discussions, and if anything even faintly resembling the application of homœopathic methods can therein be detected, I have been unable to discover it. "The favorite branches of studies" are most certainly there, *but the application of Hahnemann's methods are not there.* We find, instead of it, a rehash of allopathic proceedings which before another decade passes will be superseded by others equally pernicious. We should regard only those physicians as belonging to the consensus of the competent, who have experimented honestly, as Hahnemann demands. Those who, after such honest work, have failed to find his teachings true should disavow the name of Homœopathy. If, after honest experiments, they are better satisfied with the so-called "regular methods," let them seek their associations and affiliations among these. Those who are contented to look upon the law of similars merely as a *rule*, to be applied as it suits the caprice of each individual, who claim the right to treat their patients by any mode or means, disregarding the responsibility of assuming the name of Homœopathy, should be honest and seek their affiliations with the eclectics. Our claim is perfectly right that such practices should not pass current as homœopathic before the public. A few years ago one of this class spoke these words to the members of a bereaved family: "Nothing could have saved the patient, as both methods of treatment were applied by me during his sickness." May we not with propriety ask what kind of Homœopathy could that have been, and what kind of allopathy? A professor of materia medica and therapeutics in New York puts himself on record with the following words:

"He who in these days will not wash out with distilled water and one seventy-five thousandth grain of Corrosive Murcury a fresh case of gonorrhœa *and cure his erring brother in twenty-four to forty-eight hours* must give up the treatment of such diseases." The millennium has really arrived for "curing" this disease in New York. The professor's statistics claim that only one in a hundred gets orchitis or rheumatism after killing the terrible gonococcus, and this admirable result is not owing to the death of the germ, but to the temperature of the solution used. This is certainly a splendid showing, but unfortunately it does not succeed with the same brilliancy in other sections of the country and where the discharge does not stop in twenty-four to forty-eight hours, but goes on frequently for months in the same old way, unless the case happens by accident to be one homœopathically suited for Corrosive Sublimate, which may now and then occur. According to the Professor, the specific has been found for this disease, but we know better, as no specific exists for a *disease*, but it does exist for the patient, and that we can only find by applying the methods of Homœopathy. If this assertion of the Professor is true, why are sounds still passed to detect a possible stricture for the obstinacy of the discharge? Why is it necessary to still cut and slash the urethra so that the "*erring brother*" will be obliged to pass catheters when he is fifty years old on account of the erring doctor's methods? We are told that we blindly follow the master, which means, I suppose, that we have not sufficient discernment to discover the faults of his teachings and methods, but just shut our eyes and "go it blind."

Still, we have sufficient vision left not to "cure" a gonorrhœa (with or without sycosis) in twenty-four to forty-eight hours, according to the latest theory of the allopathic school. Up to within a very few years every chancre was cauterized, until it became evident that secondary syphilitic diseases were caused by the suppression of the primary ulcer. The ectrotic treatment of the local manifestation is being abandoned by the more thoughtful men of the old school, and so-called constitutional treatment resorted to from the beginning of the infection. This is in accordance with Hahnemann's discovery that the primary sore is nature's effort to rid the organization of the noxa, and therefore must not be locally interfered with. If left alone no constitutional syphilis will follow. Who among us has not verified this experience over and over again?

Now there is undoubtedly a germ that runs riot in the chancre, and according to our New York authority, that germ should first be strangled, for if his killing of germs applies to gonorrhœa, it must apply to every external manifestation of disease. If it were true that the cure of sycosis, syphilis, or psora was dependent upon the killing of the germ, no such diseases could be cured with dynamized drugs, which we are doing day by day, and leaving our patients in such a condition that no trace of disease can be transmitted to their progeny. We know whereof we speak, for we have had ample opportunity to observe the results of correct treatment into the second generation. Our Association correctly demands of a candidate not only an adherence to the law of similars and the single remedy, but that he should have passed that state of mind (which most beginners have to experience) in which he doubts the efficacy of a potentized drug, and in cases of failure places the blame upon the preparation of the drug, rather than upon its selection.

The full development of Homœopathy as a system of therapeutics was only reached after Hahnemann had discovered the endless divisibility of substances when treated by his methods, and when he furthermore ascertained the curative power of substances which were inert in their crude state. No man or woman should be eligible to a "*homœopathic*" Association until they have verified this fact, and are willing to fight for it, in spite of microscope or scales. Those who have not been able to do this, are not of the consensus of the competent in matters homœopathic.

Physical science, as far as it has been developed, may still be unable to explain the efficacy of potencies above the twelfth centesimal, but the experiments on living beings have piled up evidence enough to outweigh any attempt to disprove them by "scientific" dictum. If thirty years ago an electrician had told a scientist that a number of messages could be transmitted from both ends of a single wire at the same time, the scientist would have tried to demonstrate its impossibility, no matter if the electrician had proved it to his own satisfaction. Homœopathy possesses this *scientific* fact, that most substances in their crude state are transformed into useful curative agents by the process of potentizing, and Homœopathy will patiently wait till it suits *science* to explain it. If the science of therapeutics rested only on the law of similars, it would be indeed a flimsy fabric. Under this law alone any one would be at liberty to bleed, cup, purge, irritate, etc. It has all the plastic qualities of putty, and is easily made to conform to almost anything which is now practised under the shield "of the law."

Hahnemann was the first to develop the law, not the first to find it, but the first to make it useful. Its usefulness was secured by the discovery of the dynamization of drugs—a discovery of vast importance, and as yet in opposition to all accepted facts regarding "matters," that it may still require decades before the medical world will accept it. We must remember how many discoveries in the world's history have remained unaccepted for generations until the thought and insight of men had grown sufficiently to accept the revelations of nature which a genius had unveiled.

Our battle-cry must be: No Homœopathy without *Hahnemannism !*

The grand structure of Homœopathy can rest on firm foundation only by recognizing and employing the single remedy, the potentized drug, over which is spanned the arch, inscribed "Similia similibus curantur." Without trespassing further on your time, I crave your assistance in the discharge of duties you have deputed to me, and I ask your forbearance with my own imperfect qualifications.

